# Factors Associated with Psychotropic Medications Literacy among Community Pharmacists

**DOI:** 10.3390/medicina59030618

**Published:** 2023-03-20

**Authors:** Abdelrahim Alqudah, Ghaith Al-Taani, Daniel Handal, Rahaf Al Sharab, Rawan Al Shreideh, Ahmed Al-Smadi, Esam Qnais, Omar Gammoh

**Affiliations:** 1 Department of Clinical Pharmacy and Pharmacy Practice, Faculty of Pharmaceutical Sciences, The Hashemite University, Zarqa P.O. Box 330127, Jordan; 2 Department of Clinical Pharmacy and Pharmacy Practice, Faculty of Pharmacy, Yarmouk University, Irbid P.O. Box 566, Jordan; 3 Faculty of Psychology, Balqa’ Applied University, Al-Salt P.O. Box 19117, Jordan; 4 Department of Pharmaceutics and Pharmaceutical Technology, Faculty of Pharmacy, Yarmouk University, Irbid P.O. Box 566, Jordan; 5 Department of Adult Health Nursing, Princess Salma Faculty of Nursing, Al al-Bayt University, Mafraq P.O. Box 25113, Jordan; 6 Department of Biology and Biotechnology, Faculty of Science, The Hashemite University, Zarqa P.O. Box 330127, Jordan

**Keywords:** psychotropics, knowledge, community pharmacist

## Abstract

*Background and objectives*: Community pharmacists play an important role in ensuring the patient’s adherence to medications, thus achieving therapeutic outcomes. The present study had two aims: to measure the extent of knowledge that community pharmacists had about psychotropic medications and to determine the factors associated with higher knowledge scores. *Methods:* A cross-sectional design was employed, using a structured online questionnaire. The study instrument assessed demographics, general practice characteristics related to psychotropics and a battery of factual questions that assessed the knowledge of pharmacists about psychotropic medications using closed-ended responses. A total knowledge score consisting of the sum of correct responses was calculated; the passing score was 75%. A total of 676 pharmacists completed the survey. *Results:* Only 20% passed the threshold score (75%) for the factual knowledge questions, and only (11.0%) were very comfortable with their knowledge of psychotropic agents. A total of 49.0% of the respondents felt that they had been adequately trained to counsel patients on psychotropic agents. According to the regression model, pharmacists who reported higher knowledge were more experienced (0.63, (0.26–1.0), *p* < 0.001), reported studying the topic in the pharmacy school (0.77 (0.27–1.26), *p* = 0.002) holding a Doctor of Pharmacy (Pharm D) degree (0.24 (0.05–0.43), *p* = 0.01), and reported a higher perceived knowledge (0.29 (0.01–0.38), *p* = 0.038). *Conclusion:* Community pharmacists reported poor knowledge of psychotropic medications, and continuous medical and professional education programs are mandatory.

## 1. Introduction

Depression and anxiety are leading causes of disability worldwide, contributing to 2.5% of the global burden of disease [1]. It has been calculated that the global economy loses about USD 1 trillion every year in productivity due to mood disorders [2]. Furthermore, nearly 800,000 individuals worldwide commit suicide each year [3].

Jordan is experiencing a considerable surge in depression and anxiety symptoms. Large-scale cross-sectional studies carried out among adolescents and college students demonstrated alarming findings. In one study of college students, the majority reported severe anxiety symptoms (5). Another study recruited more than 2000 adolescents and revealed that 34% experienced moderate to severe depression (6). A further nationwide study (>8000 participants) reported depression in 66% of the study sample (7).

Furthermore, depression and anxiety were highly associated with physical health and chronic diseases. For instance, one recent study demonstrated that half of Coronary Artery Disease Patients suffered from depression [4], and another report found that 50% of mothers suffered from postpartum depression [5]. In addition, a school-based survey of adolescents recruiting more than two thousand participants demonstrated a decreased stigma towards depression, and a willingness to seek social or medical advice [6].

The demand for psychotropic medications is increasing globally [7]. For example, in the United Kingdom, the total number of antidepressant prescriptions was estimated to be 78 million [8]; moreover, the cost of sertraline increased by GBP 113 million in 2020 compared to 2019 [7]. In Jordan, although no published research is indicative of the demand for antidepressants, several studies have highlighted striking evidence of depression prevalence. In one study that included >8000 youth participants, 66% reported a loss of joy and 49% reported a loss of hope [9] and another study estimated depression to occur a rate of 40% among healthcare workers during the COVID pandemic [10].

Community pharmacists play an important role in ensuring the patient’s adherence to their treatment. Pharmacists should address the reasons behind nonadherence, such as the patient-related factors including forgetfulness, polypharmacy, and disease misconceptions that affect the patterns of medication use [11,12,13]. Another reason could be social factors, such as cultural matters and religion, where mental illness is perceived as a punishment or as a sign of lacking morality. In these instances, prayers or meditation are often used for the treatment of these diseases [14,15]. Yet the major reasons for nonadherence are the delayed efficacy and the side effect profile [16,17].

An increase in community pharmacy accessibility, paired with expansion, was intended to provide community pharmacists with the capacity to deliver optimal service for patients receiving psychotropic medications such as antidepressants or anxiolytics [18]. A 2021 study, which included 459 pharmacists, reported that Jordanian pharmacists felt they had insufficient knowledge and confidence with psychotropic medications [19].

Several factors are relevant to a pharmacist’s knowledge and confidence when decision-making, such as the quality of education, experiential training, years of experience, and others [20,21]

Given the clear importance of this topic, we sought to assess pharmacists using a large-scale study with a focus on technical knowledge regarding psychotropic medications.

Therefore, the current research was aimed at measuring the extent of knowledge of the community pharmacists about psychotropic medications, with the goal of then determining factors associated with higher knowledge scores.

## 2. Materials and Methods

The present cross-sectional study assessed the knowledge regarding psychotropic medications among community pharmacists in Jordan using a pretested validated, online survey.

### 2.1. Design

This is a cross-sectional study. The sampling adopted was convenience sampling that covered community pharmacists in different geographical locations in Jordan. An invitation to take the survey was provided to the pharmacist through professional online platforms, as well as invitations provided to the pharmacist in their community pharmacies via personal contact by trained students. Sample size calculation. using the online sample size calculator (www.raosoft.com accessed on 27 November 2022) with a margin of error of 5%, a confidence level of 95%, and a response distribution of 50% revealed the need for the inclusion of 367 pharmacists, based on the 8000 community pharmacists registered in Jordan.

Participation in the study was available in two ways, online and via a site visit. For the online distribution, an invitation to take part in the survey was uploaded to online platforms, including professional pharmacy social media platforms. The invitation included information about the research and why it was conducted, the approximate time needed to complete the survey, the anonymity of the data collected, that the completion is voluntary, and confidentiality and secure storage of the data, as well as a link to complete the survey using google forms software. The first page of the survey on the google form included the consent to complete the survey. The pharmacists were provided with information about the study and asked whether they agreed to take part by pressing the “I agree” button. Only those who agreed to take part would have access to the survey. To ensure the quality and completeness of the responses and to prevent duplicate entries the google forms software, settings were set to “one response” and “required”. For the distribution via community pharmacist visits, trained students entered community pharmacies in person, explained the study procedure and why it was being conducted, and asked the pharmacist to take part in the study. Only those who agreed and signed a consent form were invited to complete the google form survey. The survey collected data anonymously. The present study protocol was reviewed by the institutional review board at Al Bayt University and ethical approval was obtained to conduct this research (IRB reference number 7/2021/2022).

### 2.2. Instrument

In the present study, a systematic approach was adopted to develop and pre-test the instrument to achieve the research objectives. The instrument used in the present study was an adapted version of a previously published instrument [22] with some modifications. We carried out reliability analysis for the instrument and the Cronbach’s alpha result was 0.646, which was considered acceptable (>0.6).

The developed instrument used in the present study has been subject to face and content validity by review by faculty members who were pharmacists and hold a postgraduate degree in pharmacy. After development, the survey was distributed to 10 pharmacists as a pilot distribution to assess the clarity of the items used, usability, and functionality, particularly with online distribution. Comments raised from the faculty members’ review and pilot distribution were addressed and minor changes to the developed survey were made.

The instrument utilised consisted of 26 items, including (1) demographic and practice characteristics, e.g., age, sex, and type of pharmacy; (2) general practice characteristics related to antidepressants, such as perceived knowledge of antidepressants, number of antidepressant prescriptions dispensed, and training received concerning antidepressants; and (3) a battery of factual questions that assess pharmacists’ knowledge about psychotropic medications (antidepressants/anxiolytics), pharmacology and therapeutics, using closed-ended responses, in most of the questions as yes, no, and unsure. A total knowledge score consisting of the sum of correct responses was calculated. Raw data were transformed from the Google form into an Excel sheet, cleaned, and analysed.

### 2.3. Data Analysis

Standard statistical methodologies were used to assess the knowledge of community pharmacists about antidepressants. The statistical package for social sciences (SPSS) was used to run the analysis. Descriptive statistics, e.g., means and frequencies, were used to summarise the data. Independent sample t-test and one-way ANOVA were used to compare the mean knowledge score by different demographic and practice variables. The variable that had the potential (*p* < 0.1) to be associated with an increased total knowledge score was entered into a multivariate linear regression analysis to identify variables that had independent, statistically significant predictors associated with an increased total knowledge score. The *p*-Value was set at *p* ≤ 0.05.

## 3. Results

### 3.1. The Study Sample Characteristics

The number of approached pharmacists was 752; however, a total of 676 pharmacists completed the survey, of which about three-quarters were females. The most common age group of respondent pharmacists was 20–29 years (78.0%), followed by the age group 30–39 years (14.1%). Less than 10 were more than 40 years of age. In Jordan, there are two first university degrees in pharmacy, namely BSc and Pharm D. Most respondents had a BSc degree (78.8%) and 13.0% had a Pharm D. It is worth mentioning that about 7% had a master’s degree. Respondents were distributed about the work setting (chain or independent pharmacy) and schedule. One third (30.4%) of the respondent community pharmacists were freshly graduated, whereas approximately 40% had less than five years’ experience. Full details of these variables are summarised in Table 1.

### 3.2. The Perceived Knowledge of Psychotropics

Table 2 summarizes the perceived knowledge of psychotropics of respondents. Most commonly, 71.8% of the respondent community pharmacists dispensed 50 prescriptions or less of antidepressants per month. Just 11.0% of the respondent community pharmacists were very comfortable with their knowledge of antidepressant/anxiolytic agents. About half (49.0%) of the respondent community pharmacists felt that they had been adequately trained to counsel patients on antidepressant/anxiolytic agents. Only 10.7% of the respondent community pharmacists strongly agreed that they had adequate education about antidepressant/anxiolytic agents during their pharmacy training. Regarding the educational approaches used in pharmacy school, about half of the respondents received experiential rotation in psychiatry and 13.0% did not learn about antidepressant/anxiolytic agents in pharmacy school.

### 3.3. The Actual Knowledge of Psychotropics

Table 3 presents the responses to the actual knowledge of psychotropic agents. The highest frequency correct answer of the individual knowledge statement was 66.5% and was for the use of antidepressants for generalised anxiety disorders. Less than 40% of correct responses were reported for the following knowledge statements, including patients get easily addicted to antidepressants (40.1%), the use of selective serotonin reuptake inhibitors is useful in treating pain (37.5%), length of treatment of benzodiazepines (36%), selective serotonin reuptake inhibitors efficacy compared with tricyclic antidepressants (25.9%), and treating anxiety disorders with benzodiazepines (25.3%).

### 3.4. Comparing the Knowledge Mean Scores across the Study Variables

Table 4 compares the mean knowledge score by different variables using inferential statistics. Statistically significant differences were achieved as follows: males achieved higher knowledge scores, those working in chain pharmacies achieved higher scores, those who had learned about antidepressants/anxiolytics in pharmacy school achieved higher scores, highest knowledge scores were achieved from community pharmacists aged 30–39 years, Pharm D holders had the highest knowledge scores than those graduates from other disciplines, those who dispensed 50 prescriptions or fewer of antidepressants achieved highest knowledge scores, those who reported being comfortable with their knowledge of antidepressant/anxiolytic agents achieved the highest knowledge score, those who felt that they had been adequately trained to counsel patients on antidepressant/anxiolytic agents have the highest scores and those who agreed to the statement that they received adequate education about antidepressant/anxiolytic agents during their pharmacy training achieved the highest knowledge scores.

### 3.5. Predictors of Higher Actual Knowledge Scores of Psychotropics

An initial univariate analysis was carried out to assess the candidate variables. Candidate variables to be included in the model should have had plausible trend of association with the outcome variable and had a trend of statistical significance for the outcome variable on univariable linear regression analysis (i.e., *p* < 0.1) (Table 5).

Afterwards, independent predictors that were included in the multivariate linear regression model for the total knowledge scale are illustrated in Table 5. Experienced community pharmacists, i.e., not fresh graduates, lower prescriptions served, learned about antidepressants/anxiolytics in the pharmacy school, having a higher degree (including Pharm D), and being comfortable with their knowledge of antidepressants/anxiolytics were associated with increased knowledge scores (Table 6).

## 4. Discussion

This study reported that community pharmacists have a poor knowledge regarding psychotropic medications. Pharmacists who reported higher knowledge were more experienced, reported studying the topic in the pharmacy school, held a Doctor of Pharmacy (Pharm D) degree, and reported a higher perceived knowledge.

Pharmacists’ knowledge is a key factor for effective patient engagement, which leads to success in the therapeutic plan, especially in chronic diseases [23,24]. For example, a longitudinal study demonstrated that the effect of the pharmacist telemonitoring of antidepressants showed a significant and positive effect on patients’ feedback, knowledge, experience, and medication beliefs [25].

Our findings are consistent with the previous literature demonstrating poor knowledge of psychotropic medications. In one study, about 40% of pharmacists admitted to not being involved in antidepressant counselling, and 36% of pharmacists admitted to discussing the antidepressants’ side effects with only a few patients [26]. Furthermore, in one study, less than 20% of patients initiated on antidepressants were educated about the possible side effects of antidepressants, and only 34% of the pharmacists mentioned the need to continue the antidepressant for at least six months [27].

Although this poor knowledge is reflected in poor patient-related outcomes and satisfaction, nevertheless, no published studies coming from Jordan have related the pharmacist’s role to patient satisfaction.

In our study, pharmacists with higher experience and the Pharm D participants achieved better knowledge compared to the BSc programme graduates. Our findings support previous studies; the pharmacists’ years of experience were reflected in their knowledge and confidence [28]. Furthermore, this finding can be explained by the fact that the years of experience are related to the accumulated knowledge acquired through continuous medical education programmes, interaction with other healthcare professionals, and the number of prescriptions dispensed [29,30].

The findings of this study revealed that Pharm D participants achieved superior knowledge. Our findings support previous studies demonstrating that Pharm D graduates are more knowledgeable compared to bachelor’s degree graduates [31]. The Pharm D programme comprises more detailed theoretical courses and one-year experiential training courses applied in hospitals and community pharmacies. According to the literature, the interventions of clinical pharmacists, including drug monitoring, patient education, and drug management have a positive effect on mental health problems [32].

The study has several strengths, such as the objective measurement of the actual knowledge of the most commonly used psychotropic agents, the sample size, and the diversity of the participants recruited. However, the online data collection approach could have led to inaccurate data collection compared to the site visit. Additionally, the pharmacist’s knowledge was not reflected in their role in patient adherence and improvement. No data are available about how much pharmacists influenced adherence to psychotropic agents. Moreover, data are missing about the pharmacist’s role in the management of mental health outcomes in Jordan.

## 5. Conclusions

To conclude, Jordanian community pharmacists reported poor knowledge of psychotropic medications. Implementing Pharm D programmes, and enhancing the pharmacist’s experience are crucial factors in improving the knowledge of psychotropic medications. It is the duty of teaching institutions and the Jordanian Pharmacists Association to monitor and improve the quality of the community pharmacy service of psychotropic education.

## Figures and Tables

**Table 1:** Demographic and practice characteristics of respondent pharmacists.

Variable		Frequency	Percent
Age (year)	20–29	527	78.0
	30–39	95	14.1
	40–49	33	4.9
	50–59	18	2.7
	≥60	3	0.4
Sex	Female	523	77.4
	Male	153	22.6
Highest academic degree attained	Bsc.Pharmacy	533	78.8
	Bsc.Pharm D	88	13.0
	Masters	47	7.0
	Doctoral	8	1.2
Work setting	Chain pharmacy	181	26.8
	Independent pharmacy	495	73.2
Working schedule	Alternating	135	20.0
	Evening shift	166	24.6
	Owner	62	9.2
	Morning shift	313	46.3
Length of pharmacy practice	Freshly graduated	205	30.4
	<5 years	274	40.7
	5–10 years	120	17.8
	11–20 years	47	7.0
	>20 years	28	4.2

**Table 2:** Psychotherapeutics knowledge perception of respondent pharmacists.

Variable		Frequency	Percent
Number of prescriptions or refills on antidepressants and anxiolytics you serve per month	50 or less	484	71.8
	51–100	78	11.6
	101–500	21	3.1
	More than 500	16	2.4
	Not Applicable	75	11.1
Do you feel comfortable with your knowledge of antidepressant/anxiolytic agents?	Very Comfortable	74	11.0
	Comfortable	190	28.1
	Neutral	316	46.8
	Uncomfortable	83	12.3
	Very Uncomfortable	12	1.8
Do you feel that you have been adequately trained to counsel patients on antidepressant/anxiolytic agents	Yes	331	49.0
	No	160	23.7
	I don’t know	185	27.4
Did you complete an experiential rotation in psychiatry while in pharmacy school?	Yes	327	48.4
	No	349	51.6
Did you learn about antidepressant/anxiolytic agents in pharmacy school?	Yes	587	87.0
	No	88	13.0
I have received adequate education about antidepressant/anxiolytic agents during my pharmacy training.	Strongly Agree	72	10.7
	Agree	260	38.5
	Neutral	236	35.0
	Disagree	91	13.5
	Strongly Disagree	16	2.4

**Table 3:** The distribution of the actual knowledge answers of the respondents.

Knowledge Statement	Correct Answer	Correct		I’m Not Sure	
		Frequency	Percent	Frequency	Percent
The maximum length of time a benzodiazepine should be used	2 weeks	243	36.0	133	35.3
The recommended treatment for anxiety disorders is currently through benzodiazepines	False	171	25.3	109	16.1
Etifoxine is pharmacologically classified as	Anxiolytic	316	46.8	-	-
People who are appropriately treated with antidepressants are less likely to commit suicide than those who are depressed and are on no treatment	True	411	60.9	103	15.3
Are all antidepressants useful and approved for the management of depression in children and adolescents	False	442	65.5	97	14.4
Treating Generalised anxiety disorder is done by using antidepressants (SSRIs or SNRIs)	True	449	66.5	101	15.0
For the treatment of Neuropathic pain, duloxetine is used	True	398	59.1	134	19.9
Selective-Serotonin-Reuptake-Inhibitors, like Sertraline, are useful in treating pain	False	253	37.5	107	15.9
Patients can get easily addicted to antidepressants (SSRIs, SNRIs)	False	271	40.1	102	15.1
Using antidepressants such as SSRIs is more effective clinically than using Tricyclic antidepressants	False	174	25.9	156	23.2
If treatment with an antidepressant fails, the patient can be shifted to another drug from the same class.	True	309	45.8	117	17.4
A patient on an antidepressant can feel improvement within 6–8 days.	False	365	54.1	113	16.7

**Table 4:** Differences in the knowledge mean scores across the study variables.

Variable		Mean Knowledge Score	*p*-Value
Sex	Female	5.5425	0.033
	Male	5.9803	
Work setting	Chain pharmacy	5.9609	0.025
	Single pharmacy	5.5255	
Experienced?	Fresh graduate	5.1244	<0.001
	Experienced	5.8565	
Did you learn about antidepressant/anxiolytic agents in pharmacy school?	Yes	5.7427	0.002
	No	4.9535	
Age (years)	20–29	5.4933	0.011
	30–39	6.3579	
	40–49	5.6452	
	50–59	6.0556	
	≥60	6.3333	
Highest educational level	Bsc.Pharmacy	5.4231	<0.001
	Bsc.Pharm D	6.6477	
	Masters	6.2553	
	Doctoral	5.3750	
Number of prescriptions or refills on antidepressants and anxiolytics you serve per month	50 or less	5.8672	0.001
	51–100	5.0526	
	101–500	4.8095	
	More than 500	5.2500	
	Not Applicable	5.0959	
Do you feel comfortable with your knowledge of antidepressant/anxiolytic agents?	Very Comfortable	5.6216	0.002
	Comfortable	6.1011	
	Neutral	5.5446	
	Uncomfortable	5.1605	
	Very Uncomfortable	4.2500	
Do you feel that you have been adequately trained to counsel patients on antidepressant/anxiolytic agents	Yes	5.8723	0.001
	No	5.7673	
	I don’t know	5.1154	
I have received adequate education about antidepressant/anxiolytic agents during my pharmacy training.	Strongly Agree	5.0972	0.019
	Agree	5.8217	
	Neutral	5.7296	
	Disagree	5.5333	
	Strongly Disagree	4.3750	

The difference in the knowledge means scores across the study significant variables using t-test and one-way ANOVA. *p* < 0.05 is considered significant.

**Table 5:** Univariate analysis for candidate variables.

Variable with Plausible Relationship	*p*-Value on Univariable Linear Regression Analysis
Sex	0.033
Work setting	0.025
Experienced?	<0.001
Did you learn about antidepressant/anxiolytic agents in pharmacy school?	0.002
Age (years)	0.018
Highest educational level	0.001
Number of prescriptions or refills on antidepressants and anxiolytics you serve per month	0.001
Do you feel comfortable with your knowledge of antidepressant/anxiolytic agents?	0.002

**Table 6:** Predictors of higher knowledge scores.

Variable	B	95% C.I. for OR	*p*-Value
Experienced?	0.63	0.266–1.000	0.001
Number of prescriptions or refills on antidepressants and anxiolytics you serve per month	−0.22	−0.345–−0.092	0.001
Did you learn about antidepressant/anxiolytic agents in pharmacy school?	0.77	0.273–1.260	0.002
Highest educational level	0.24	0.057–0.431	0.010
Do you feel comfortable with your knowledge of antidepressant/anxiolytic agents?	0.20	0.011–0.385	0.038

The final variables included in the linear regression model for knowledge of psychotherapeutics. Higher scores were defined as correctly answering 75% of the questions. aOR: adjusted odd ratio.

## Data Availability

The data presented in this study are available on request from the corresponding author.
